# A brief overview of SARS-CoV-2 infection and its management strategies: a recent update

**DOI:** 10.1007/s11010-023-04848-3

**Published:** 2023-09-24

**Authors:** Alakesh Das, Surajit Pathak, Madhavi Premkumar, Chitra Veena Sarpparajan, Esther Raichel Balaji, Asim K. Duttaroy, Antara Banerjee

**Affiliations:** 1 Faculty of Allied Health Sciences, Chettinad Hospital and Research Institute (CHRI), Chettinad Academy of Research and Education (CARE), Kelambakkam, Chennai, Tamil Nadu 603103 India; 2 Department of Nutrition, Faculty of Medicine, Institute of Basic Medical Sciences, University of Oslo, Oslo, Norway

**Keywords:** SARS-CoV-2, Inflammatory responses, Cytokine storm, Immunopathology, Re-infection, Therapy

## Abstract

The COVID-19 pandemic has become a global health crisis, inflicting substantial morbidity and mortality worldwide. A diverse range of symptoms, including fever, cough, dyspnea, and fatigue, characterizes COVID-19. A cytokine surge can exacerbate the disease’s severity. This phenomenon involves an increased immune response, marked by the excessive release of inflammatory cytokines like IL-6, IL-8, TNF-α, and IFNγ, leading to tissue damage and organ dysfunction. Efforts to reduce the cytokine surge and its associated complications have garnered significant attention. Standardized management protocols have incorporated treatment strategies, with corticosteroids, chloroquine, and intravenous immunoglobulin taking the forefront. The recent therapeutic intervention has also assisted in novel strategies like repurposing existing medications and the utilization of in vitro drug screening methods to choose effective molecules against viral infections. Beyond acute management, the significance of comprehensive post-COVID-19 management strategies, like remedial measures including nutritional guidance, multidisciplinary care, and follow-up, has become increasingly evident. As the understanding of COVID-19 pathogenesis deepens, it is becoming increasingly evident that a tailored approach to therapy is imperative. This review focuses on effective treatment measures aimed at mitigating COVID-19 severity and highlights the significance of comprehensive COVID-19 management strategies that show promise in the battle against COVID-19.

## Introduction

Severe acute respiratory syndrome coronavirus 2 (SARS-CoV-2), the coronavirus that caused severe acute respiratory syndrome’s deadly breakout in 2019, ravaged the entire globe by causing the death of over 4 million people. The coronavirus that caused coronavirus disease 2019 (COVID-19) has a mortality rate of approximately 2% [1, 2]. When infected by the virus, one acquires a high chance of being prone to acute respiratory distress syndrome (ARDS) [3]. The disease spread all around the globe after the local spread that took place within China [4]. The World Health Organization (WHO) announced the virus outbreak as a global pandemic on 30 January 2020 and labeled the disease as COVID-19. Asymptomatic patients were unaffected by organ failures or any inflammatory problems that can infect healthy people [4–6]. The most common symptoms seen in infected patients are fever, sore throat, cough, and body pain. Initially, it was noted that patients show symptoms such as respiratory troubles and chills all over the body [7]. Statistics show that children are often asymptomatic. It is assumed that it could be due to many natural killer (NK) cells. Adverse cases of infection are usually seen in the old or the elderly population [8]. Studies show that the prevalence of the virus in children was approximately 2.3%, and the mortality rate among children was around 0.2%.

In contrast, no mortality cases were reported in children below 9 years of age [9]. The disease frequency tends to increase with a person’s age or population. Females show slightly higher vulnerability than men. The frequency of the disease in the elderly population was recorded to be around 11.49% [10]. The virus spreads among people through close contact. Aerosols are found to be a significant route of transmission. The virus proliferates in the epithelial cells of the mouth and lowers respiratory tract shedding for about 3 to 5 days instead of 5 to 7 days [11].

**Fig. 1:**
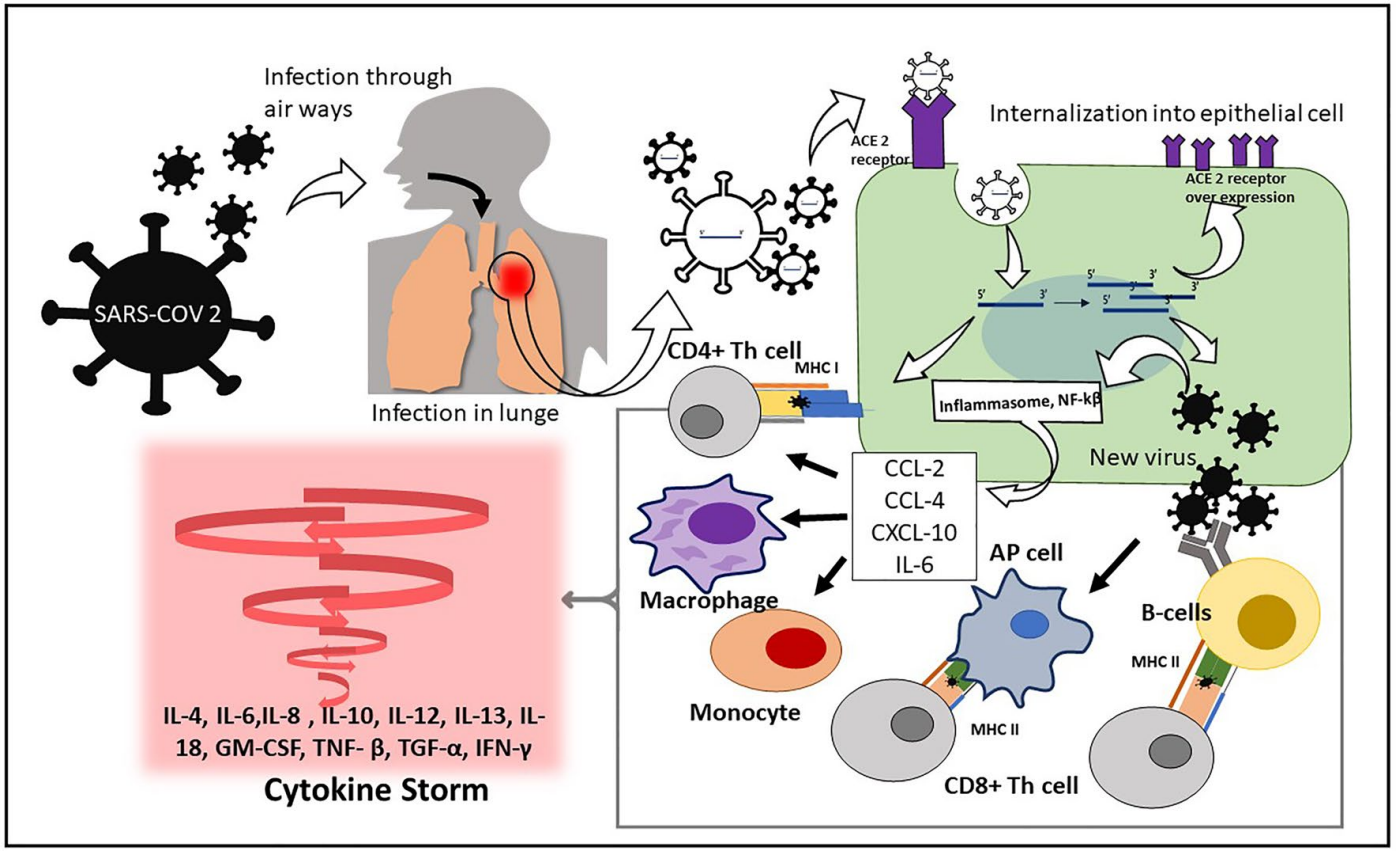
Diagram representing the SARS-CoV-2 infection in humans and its immune evasion leading to cytokine storm

The action of the virus starts with the viral replication in the lower respiratory tract region, followed by the attack on organs like the kidney and gastrointestinal (GI) tract since these organs express angiotensin-converting enzymes 2 (ACE2) [12]. The virus then causes injury to the immune-mediated mechanisms by causing inflammation [13]. The infection results in decreased lymphocyte count and increased neutrophil count. Usually, a significant drop in CD8^+^ T-cell levels was seen in critical patients [12]. Today, COVID-19 is diagnosed using RT-PCR. Hydroxychloroquine is the drug used to treat COVID-19 [13]. Remdesivir is a widely used medication for the management of COVID-19 [14]. In recent studies, elevated cytokine levels were noticed in the plasma of patients [15]. Cytokines are responsible for releasing interferons (IFNs), interleukins (ILs), and several tumor necrosis factors (TNFs) [16]. This surge causes damage to the host cells causing an inflammatory immune response in patients. A cytokine storm, which denotes the release of an excessive quantity and great abundance of cytokines, is the term used to describe the magnitude of an unbounded cytokine network state. Malfunctions of cytokines in certain diseases can result in a fluctuation in the cytokine network and clinical manifestations Fig. 1. The accumulating cytokines form a storm, which aids in the spread of SARS-CoV-2. As a result, cytokines are released more often, triggering a hostile inflammatory response. We have previously discussed recent discoveries concerning COVID-19 infections within an epigenetic framework and have outlined potential future directions involving epigenetic drugs for therapeutic interventions. The utilization of epigenetic markers associated with COVID-19, its comorbidities, and various stages of infection holds the potential to facilitate prompt diagnosis and the development of therapeutic interventions aimed at mitigating the severity of COVID-19 and associated mortality rates. The discovery of epigenetic mechanisms governing viral pathogenicity presents a promising avenue for the development of epi-drugs as a potential treatment strategy in combating COVID-19 [6]. This review is focused on assessing the management strategies designed to alleviate the impact of COVID-19 while underscoring the importance of holistic strategies for infective stages and post-COVID-19 management to achieve favorable outcomes The summarized research outcomes by other researchers and clinicians discussed in this review may help researchers to get an update regarding ongoing research on COVID-19 and its management strategies employed by the clinicians.

## The common immunopathology and pathway of cytokine surge associated with COVID-19 infection

The ongoing analysis focuses on understanding the cytokines responsible for triggering COVID-19 immunopathology, as well as determining the onset of cytokine storm (referred to as COVID-CS) during coronavirus infection. The damage inflicted by SARS-CoV-2 infection on immunological or epithelial cells leads to tissue damage and facilitates the release of inflammatory cytokines and chemokines. These substances subsequently initiate inflammation, giving rise to various complications. [17]. These inflammatory cytokines are responsible for lining up innate cells like macrophages, NK cells, monocytes, and neutrophils. They are in charge of activating adaptive immune cells like clusters of differentiation 8 (CD8^+^) and clusters of differentiation 4 (CD4^+^) cells [18]. The excessive production of systemic cytokines results in erythro-phagocytosis and macrophage activation, leading to disseminated intravascular coagulation (DIC), thrombosis, capillary leakage, and anemia [19]. Toll-like receptors (TLRs), NOD-like receptors (NLRs), retinoic acid-inducible gene I (RIG-I) receptors, and melanoma differentiation-associated protein 5 (MDA5) that sense the invasion of SARS-CoV-2 are immediately activated when inflamed or apoptotic cells emit reactive oxygen species (ROS) and viral proteins [17]. Type I interferons (IFN-1) are produced due to the downstream transcription factor interferon regulatory factor (IRF) 3/7 being activated, which leads to paradoxical hyperinflammation and the production of pro-inflammatory cytokines. It takes the activation of the nucleotide-binding domain, leucine-rich–containing family, pyrin domain–containing 3 (NLRP3) inflammasomes for mature IL-18 and IL-1 to be produced. Pro-inflammatory cytokines such as interleukin- (IL)-6, IL-2, tumor necrosis factor (TNF), and IFN-γ work by binding to their receptors to initiate janus kinase/signal transducers and activators of transcription (JAK-STAT) or NF-κB signaling, which boosts the release of pro-inflammatory genes and pushes up the cytokine storm (CS) threshold [20].

## Inflammatory cytokines associated with COVID-cytokine storm

IL-1β was recently revealed among the elevated cytokine levels and was employed to categorize COVID-19 patients as mild, moderate, or severe [21]. In reaction to viral RNA recognition by innate immune receptors that consist of TLRs and nuclear factor-ĸB (NF-ĸB), it is stimulated to produce numerous cytokines, such as TNF and ILs [22]. Several studies have shown that IL-1β serves as one of the primary causes of pulmonary inflammation and injury during viral infections [23]. Following SARS-CoV-2 recognition, the release of IL-1β in its pro form was triggered by caspase-1 to its mature form; the mature form binds to its target receptor IL-1R and initiates subsequent signaling processes comprising the phosphorylation of NF-ĸB transcription factor, c-Jun N-terminal kinase (JNK), and P38 mitogenic kinase [24]. These stimulated pathways will interact to promote the expression of IL-1-specific genes, which includes the vital cytokine IL-6 [25]. Monocytes, macrophages, and dendritic cells (DCs) are the primary producers of IL-6, a powerful activator of the JAK/STAT3 pathway [26]. IL-6 is a primary cytokine that promotes acute inflammation [25]. It is regulated by several cytokines and pathways, comprising IL-1β and TNF-α [27]. It has been noted that IL-6 levels are significantly elevated in COVID-19 individuals and are directly correlated with disease severity. IL-6 is indispensable for the cytokine cascade. IL-6 utilizes the well-known cis and trans-signaling pathways via the sIL-6R and mIL-6R. Only immune cells exhibiting mIL-6Rs and IL-6 bind to form the IL-6/IL-6R/ glycoprotein 130 (gp130) complex and activate downstream JAK/STAT3, MAPK, and Akt/mTOR signaling in trans-signaling. Circulating IL-6 binds to the gp130 dimer, expressed on almost all cell types, after forming complexes with IL-6Rs. Once both pathways are activated, a cascade of events culminating in activating the JAK-STAT and AKT/PI3K pathways is initiated [27, 28]. The JAK-STAT3 pathway is consequently activated in cells lacking mIL-6R expressions, such as vascular smooth muscle cells (VSMCs) and endothelial cells. It positively affects the Th17 cell differentiation, inflamed tissues and discharge of different adhesion factors and cytokines, and activation of the NF-κB pathway, developing a positive feedback loop for its synthesis [26].

Moreover, IL-6 is utilized in various biological processes, including T-cell clonal expansion, B-cell differentiation, acute inflammatory response, and mitochondrial activity [27]. The suppression of differentiation and stimulation of numerous T-helper cells promoting the production of an extensive stream of cytokines and chemokines is an additional method to mitigate cytokine release. The JAK-STAT cascade is one of the major cellular pathways responsible for regulating T-helper activation [29]. Macrophages and monocytes of the innate immune system produce most of the IL-1. IL-1 induces pro-inflammatory responses that attract immune cells and promote the synthesis of secondary cytokines, resulting in acute phase reactivity. A prolonged but substantial IFN-1 response promotes an upsurge of monocytes and macrophages and the production of pro-inflammatory cytokines in BALB/c mice afflicted with SARS-CoV, resulting in lethal pneumonia, vascular leakage, and insufficient T-cell responses. Mice infected with SARS-CoV-2 and displaying the human ACE2 entry receptor demonstrated that IFN-1 promotes inflammation. It proved that IFN-1 responses are required to recruit pro-inflammatory macrophages and monocytes to infected airways employing mice deficient in an IFN-I response, such as Ifnar/ mice or Irf3/Irf7/mice [25, 26, 29, 30].

**Fig. 2:**
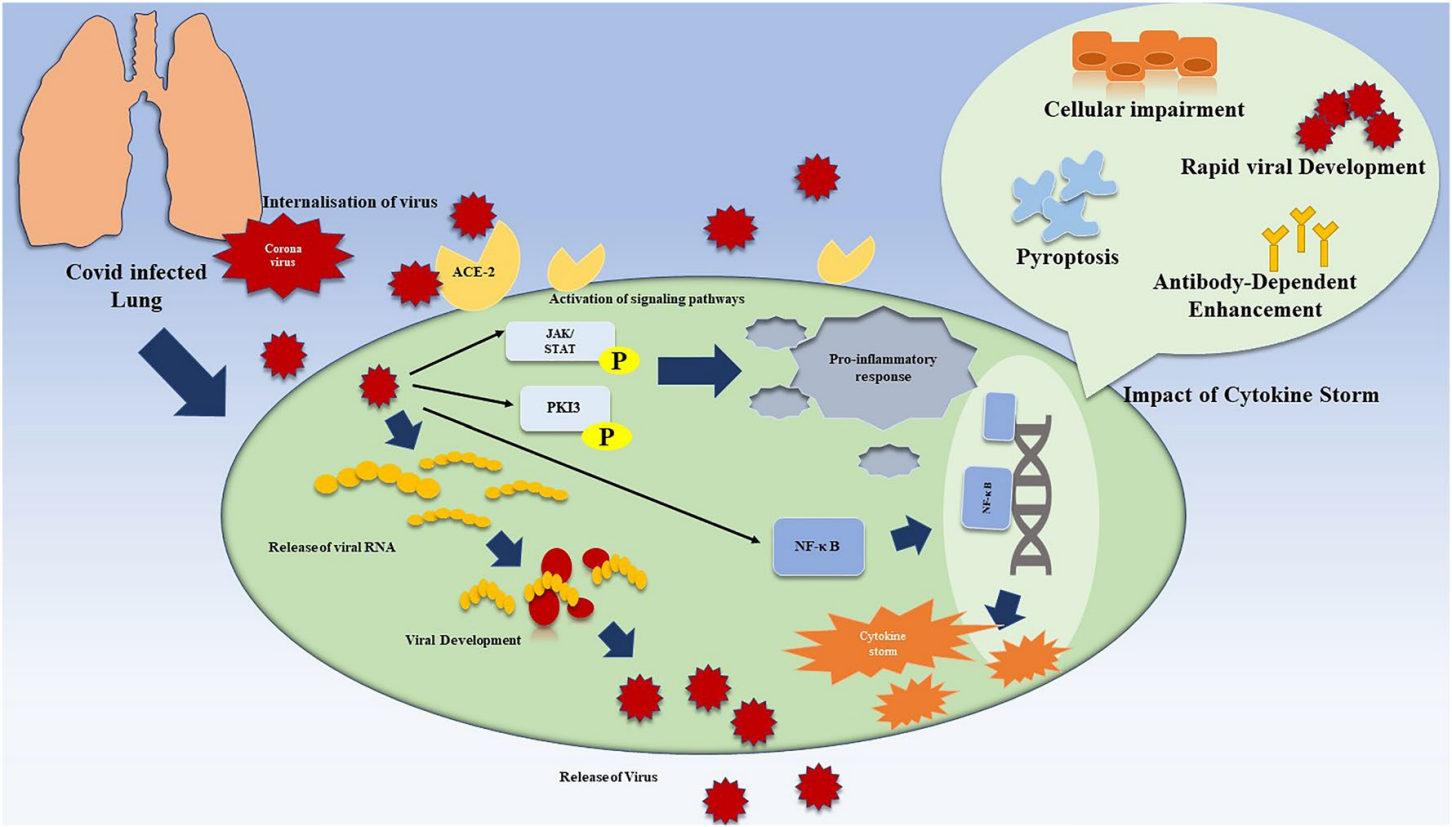
Diagrammatic representation showing the rapid viral development of SARS-CoV-2 and its role in the regulation of crucial signaling pathways associated with activating the CS

Further, TNF-α is a widely recognized cytokine that promotes inflammation and has been linked to several autoimmune, cancerous, and viral diseases. T cells, macrophages, and monocytes primarily synthesize it. Due to the presence of TNFR1 and a variety of intermediate adapters, TNF-α, the primary cause of NF-κB activation, may activate the NF-κB signaling pathway and increase the production of pro-inflammatory genes [31]. IL-2, mainly secreted by CD4^+^ cells, is essential for the proliferation and differentiation of CD8^+^ T cells, NK cells, CD4^+^ T cells, and other cells [32]. Elevated IL-2 levels were observed in numerous cases of coronavirus infections. Additionally, it was shown that COVID-19 patients had significant concentrations of IL-2 or IL-2R. The primary source of IL-2 is CD4^+^ T cells; hence, the reduced lymphocyte count during the CS stage after COVID-19 infection may at least partially underlie the declining IL-2 levels and signaling [33]. In the context of peripheral homeostasis, it is seen that T cells exhibit survival, persistence, and differentiation. The signaling pathway of interleukin-7 (IL-7) is of utmost importance. The levels of IL-7 in patients diagnosed with COVID-19 have been shown to be elevated, prompting current investigations on their association with the severity of the illness [34]. CD8^+^ T cells, B cells, DCs, macrophages, NK cells, Th2 cells, Tregs, and CD8^+^ T cells produce IL-10. Several immune cells receive signals from this interleukin through the IL-10R/JAK/STAT3 pathway. By either directly lowering macrophage and DC innate immune functions in a paracrine and autocrine way or by indirectly promoting Treg production, IL-10 reduces inflammation [32]. A negative feedback mechanism called excessive IL-10 production is used to balance out the immune system’s overactivity. However, in COVID-CS, the fine-tuning function of IL-10 is particularly insufficient during high inflammatory mediator release and pro-inflammatory cell activation. Therefore, it can be inferred that IL-10 can cure COVID-19 ARDS [35]. IL-12, a multifaceted immunoregulatory factor largely generated by B-lymphocytes, macrophages, and DCs, promotes Th1 and Th17 cell proliferation and NK cell cytotoxicity. A positive feedback loop is seen during interferon-gamma (IFN-γ) expression in macrophages, NK cells, Th1 cells, and DCs. As a result, by promoting immune cell activation, IL-12 plays a combative function in CS [34]. The primary sources of IFN-γ are NK cells, macrophages, and T cells. It plays a crucial role in immunological processes like inflammation. The activation of the JAK1/JAK2 complex takes place by holding a key role in bacterial and downstream signal transducer and activator of transcription 1-interferon- (STAT-1-IFN)-activated site cascades [31]. The significant involvement of IFN-γ in various CS-related diseases has been shown via observations of primary hemophagocytic lymphohistiocytosis (HLH) patients [36]. Figure 2 represents the rapid viral development of SARS-CoV-2 and its role in regulating crucial signaling pathways associated with activating the CS.

## Cytokines and chemokines associated with COVID-19

The genes accounting for various chemokines, including chemokine (C–C motif) ligand 2 (*CCL2),* chemokine (C–C motif) ligand 3 (*CCL3),* C-X-C motif chemokine ligand 10 (*CXCL10)*, and C–C motif chemokine ligand 4 (*CCL4)*, were over-expressed in the peripheral blood mononuclear cells [37]. Similarly, studies have also discovered that people who displayed over-expressing genes encoding for *CCL7, CCL4*, *CCL3*, and *CCL2* (and eventually the cytokines *IL-1, IL-6, IL-2, IL-10, IL-12, TNF, IFNs*) show the presence of lung macrophages [38]. The early research indicated that the vicious circle that characterizes the CS would be significantly influenced by the amount and kind of cell invasion [39].

### Cytokine storm

It has been discovered that a variety of medications, infections, malignancies, autoimmune diseases, and monogenic disorders have the potential to elicit or initiate a CS. This phenomenon is characterized by an excessive presence of cytokines in the bloodstream and increased activation of immune cells, resulting in a potentially fatal systemic inflammation. It was formerly referred to as an influenza-like condition [40]. During viral infections, aberrant active stimulation of several immune cells can disrupt the body’s natural physiologic equilibrium of pro-inflammatory and anti-inflammatory cytokines [41]. A cytokine storm induces a “suicide attack” that not only aids in the destruction of harmful microorganisms but also causes organ damage. Pro-inflammatory cytokines may repeatedly drive other immune cells development in a virtuous cycle [42].

With COVID-19, the cytokine storm is only seen in affected individuals with serious illnesses. In instances of severe SARS-CoV-2 infection, the occurrence of a CS results in a robust immune response that attacks the body, leading to the development of multiorgan failure and acute respiratory distress syndrome (ARDS), ultimately resulting in mortality [43]. While the precise etiology of ARDS in persons afflicted with COVID-19 remains uncertain, one prominent factor implicated in its pathogenesis is the upregulation of pro-inflammatory cytokines [44]. CS can also result from well-known medical techniques such as adoptive T-cell treatments, chimeric antigen receptor T-cell (CAR-T) therapy, monoclonal antibody medication regimens, and immune checkpoint-blocking inhibitors that can induce or trigger CS [45]. There have been no exact or fixed treatment recommendations for COVID-19 have been issued to date, but recent studies have demonstrated that cytokine blockades that specifically target cytokines, such as interleukin IL-1 and IL-6, offer optimistic therapeutic promise for COVID-19 [46]. Cytokines serve as signaling molecules that help define, intensify, and regulate the immune system response and can have systemic effects if they reach to a sufficient levels. Increased amounts of cytokines will influence many physiological systems in illnesses with systemic involvement [47]. A CS impacts critical COVID-19 patients, resulting in various clinical abnormalities and immune dysfunction.

## Effects of COVID-19 on the respiratory system and their lasting repercussions

Based on a prior report, thirty-three cases (30%) exhibited aberrant computed tomography (CT) findings, and seventeen patients (15.5%) displayed damaged carbon monoxide diffusion capacity (DLCO) at six months following recovering from severe acute respiratory syndrome (SARS). A study examined 97 individuals who had recovered from SARS and determined aberrant CT results, and DLCO continued to exist post one year of the infection. A retrospective, multi-center cohort study reported that an elevated percentage of COVID-19-infected individuals were found to have persistent anomalies on chest CT scans 3 months post-discharge. In the follow-up investigations of SARS patients undergoing rehabilitation, diminished respiratory function could persist for months or even years [48–51]. An elevated D-dimer level has been documented as a significant diagnostic observation in COVID-19 patients requiring further treatment. Numerous investigations have demonstrated that D-dimer at admittance predicts in-hospital mortality in COVID-19 patients. Research has shown that notable radiographic and physiological variations persisted in a substantial proportion of COVID-19 patients three months following discharge. Anti-SARS-CoV-2 IgG antibody has disappeared in several patients. To identify and appropriately treat any chronic or emergent long-term sequelae in the radiological and physiological regions, it is essential to continue following up with these patients, conducting an in-depth evaluation, and prompt rehabilitation exercises [48, 52, 53].

### Clinical abnormalities during an increase in cytokine storm in COVID-19 patients

The term CS is defined as a group of immune dysregulation illnesses marked by constitutional manifestations, inflammatory responses, and multiple organ dysfunction, all of which can progress to multiple organ failures if not appropriately treated [40]. Almost all affected individuals with CS may feel feverish [54]. Fatigue, headache, rash, anorexia, diarrhea, arthralgia, muscle stiffness, and sometimes neuropsychiatric abnormalities are also possible. These symptoms might be induced by cytokine-induced tissue destruction, or they can also sometimes be induced by immune-cell-mediated responses. Many patients suffer ARDS and hypoxemia, common respiratory symptoms [55]. In extreme cases of cytokine storm, several other bodily dysfunctions, like organ failure, may also be seen. Cytokine release syndrome-related encephalopathy is different way to define neurologic damage linked with T-cell immunotherapy [56, 57]. Many patients also have leukocytosis, leukopenia, anemia, thrombocytopenia, and hypertriglyceridemia [40]. CS is a broad term that encompasses a wide range of illnesses. Certain critical patients may have sepsis along with cytokine storm. In some chronic diseases involving the immune system, like leukemia, acute lymphoblastic leukemia, and other, unfortunately, relying on clinical criteria is insufficient for distinguishing between sepsis-induced CS and CS resulting from chimeric antigen receptor T-cell (CAR-T cell) therapy. Individuals experiencing CS generated by CAR-T-cell treatment have moderately elevated levels of IL-1, procalcitonin, and indicators of endothelial damage in their circulation in comparison with patients with sepsis-driven CS. The latter group, however, has much higher circulating levels of IL-1 and procalcitonin. The use of IL1RA, gp130, and interferon may be employed for the prediction or assessment of the degree of cytokine release syndrome induced by CAR-T-cell therapy [58, 59].

### Prospects for vaccine escape variants of SARS-CoV-2

To entirely eliminate COVID-19, one of the key goals is the production of neutralizing antibody (nAbs), which aim at the receptor-binding domain (RBD) of the SARS-CoV-2 spike using vaccination. Analysis has shown serious concerns about the effectiveness of vaccines against SARS-CoV-2 because the virus spreads quickly and is resistant to neutralization. Thus, the E484K mutation, along with the K417N mutation to a lesser extent, was found to be mediating neutralization resistance. Pseudo viruses with either the N501Y or E484K spike mutation were only partly susceptible to neutralization. E84K/K417N refers to the specific mutations in the spike protein of the SARS-CoV-2 virus, which is responsible for the virus’s entry into human cells. This mutation involves a change in the amino acid at position 484 of the spike protein from glutamic acid (E) to lysine (K), and the K417N mutation involves a change in the amino acid at position 417 of the spike protein from lysine (K) to asparagine (N). N501Y a “pseudo virus” with the N501Y mutation, typically refers to a laboratory-created virus-like particle that incorporates the N501Y mutation in its spike protein. This mutation involves a change in the amino acid at position 501 from asparagine (N) to tyrosine (Y). Pseudo virus with the E484K/ K417N alterations was similarly resistant to neutralization by post-vaccination sera as wild-type SARS-CoV-2. However, the SA-N501Y/K417N/E484K pseudo virus was noteworthy as it was highly infectious and only moderately neutralized by vaccination sera. Increased infectivity and viral spread may arise from N501Y because of its potential to enhance the virus’ interaction with human angiotensin‐converting enzyme 2 (hACE2). When further alterations like E484K or K417N are introduced, neutralizing sensitivity is enhanced. Studies have shown that the Pfizer vaccination has minimal efficacy against SA-N501Y/K417N/E484K pseudo virions. Compared to the wild-type SARS-CoV-2 pseudo virus, the mean neutralization efficiency of vaccination sera against this pseudo virus was reduced by 6.8. In a recent study, Pfizer found that their vaccine was only slightly more effective against the SA variant than against wild-type SARS-CoV-2; this discovery is inconsistent with that finding. These findings highlight the need for increased awareness surrounding the spread of variations. There may also be a need to create new vaccinations with increased neutralizing power against certain SARS-CoV-2 subtypes [60–63].

### Immunological dysfunctions

SARS-CoV-2 infections trigger immunological responses from the entire immune system. A considerable percentage of cytokine-secreting cells are initially activated through both host defense mechanisms [64]. The pathogenicity of COVID-19 is associated with viral activation of the cytoplasmic NLRP3 inflammasome, which adds to the complication of the disease’s pathogenesis. Pro-inflammatory cytokines like interleukin IL-1 and IL-18 are produced [65]. Several innate immune signaling proteins are attacked by SARS-CoV-2 viral proteins. While Nsp13 and Orf9c target the NF-κB pathway, Nsp13, Nsp15, and Orf9b target the IFN pathway. SARS-CoV-2 Orf6 impairs NUP98-RAE1, an interferon-inducible mRNA nuclear export complex. Orf3b and Orf9c are canonical replication proteins for SARS-CoV-2 [66]. SARS-CoV-2 infects human T-cell lines via a new pathway using the CD147 spike protein found on T-cell surfaces [66]. CD147 is found in many tissues, especially in hypoxic conditions, and is implicated in cell proliferation, death, malignant motility, metastasis, and differentiation. Nearly every kind of cancer has been associated with dysregulation of CD147, which regulates activities that encourage invasion and metastasis of tumor cells [67].

IL-6 attracts CD8^+^ T cells, led by viral illness and cross-presentation by major histocompatibility complex class I (MHC class I) on dendritic cells, activate them to become cytotoxic T lymphocytes (CTLs). Interferon, a type II interferon, is released by CTLs to boost the antiviral response. Simultaneously, dendritic cells transmit major histocompatibility complex class II (MHC II) antigen complexes to naive CD4^+^ T cells, which activate them [68]. Helper CD4^+^ T cells can increase or reduce inflammation by relying on the lineages. JAKs play a function in cytokine signaling and impact how T cells evolve into Th cells. In viral infections, the most common kind of Th cell is Th1, which generates more interferon [69].

To combat infections, neutrophils emit particular leukotrienes, reactive oxygen species, and neutrophil extracellular traps (NETs). It also causes tissue damage and may cause lung damage [64]. The severity of COVID-19 phenotypes may be linked to the time of neutrophil recruitment. Lower interferon levels cause initial neutrophil recruitment to be lower. As a result of the reduced initial response, protracted immunological activation is seen. Delayed neutrophilic inflammation might affect the CS’s etiology [64]. The principal CD4^+^ T-cell offender in the cytokine storm might be Th17 cells. IL-17, a pro-inflammatory cytokine that promotes granulopoiesis and neutrophil recruitment, is abundant in Th17 cells, and an abnormal Th17 cell response is also linked to cytokine dysregulation [70]. CD4^+^ and CD8^+^ T cells get exhausted when they cannot sustain long-term activation, similar to high-grade chronic viral infections [64].

### Diagnosis of cytokine storm in COVID-19

Similar to all other viral-induced respiratory illnesses, COVID-19 primarily manifests with the symptom of fatigue [71]. A CS, which denotes the release of an excessive quantity and great abundance of cytokines, is the term used to describe the magnitude of an unbounded cytokine network state. In an individual concerned with COVID-19, it is diagnosed as the increase of IL-2 IL-7, along with other interleukins and chemokines. Malfunctions of cytokines in certain diseases can result in a fluctuation in the cytokine network and clinical manifestations [42, 72]. A retrospective study undertaken in Wuhan, China, involving 150 cases of COVID-19 found that increased ferritin (mean 1297·6 ng/ml in non-survivor vs 614·0 ng/ml in survivors; *p* < 0·001) and IL-6 (*p* < 0·0001) are indicators of mortality and serious complications, indicating an unnerving function for virally prompted hyperinflammation [73]. This cytokine profile is comparable to secondary hemophagocytic lymphohistiocytosis (sHLH), a less-recognized condition typically caused by viral infections [74]. HLH is a hyperinflammatory syndrome marked by multiorgan dysfunction and severe hypercytokinemia. As sHLH and cytokine storm syndrome share a comparable symptomatic pattern of hyperinflammation, these conditions may be classified as belonging to the same disease spectrum. It is of tremendous significance to recognize this particular group of patients, as they may be candidates for immune suppression, thus reducing morbidity and increasing survival chances. In addition to several retrieved biochemical markers of hyperinflammation, such as increased acute phase reactants, enhanced ferritin levels, and cytopenia’s cumulative scores such as H-score have been developed to assist specialists in making decisions and identifying the likelihood of sHLH [75]. The same methodology can be implemented with considerable reliability to the COVID-19 cytokine storm syndrome. Early diagnosis clinical aspects become even more complicated once the disease has triggered other pathological processes [76]. The most proposed test for analysis of SARS-CoV-2 is the nucleic acid amplification test (NAAT), which uses RT-PCR, which recognizes the virus’s genetic material from the sample extracted from nostrils. A single positive result is adequate for treatment. The secretion of the lower respiratory tract is extracted, and a serological test is done, which is also performed in symptom-free patients for diagnosis [77]. The principle for examining cytokine storm in COVID-19 includes a decline in blood lymphocyte count and elevated systematic inflammatory indicators, along with multiple cytokines, such as IL-1β, IL-6, and TNF-α [78]. Many research studies propose that diagnoses involve nucleic acid tests and fungal infection tests. Many critical COVID-19 cases undergo CS surge [79]. Other diagnostic features are acute inflammatory symptoms and organ dysfunction caused by inflammation. In critical COVID-19 patients, it is vital to restrict the lethal effect of this particular IL-6. One of these molecules, which acts as an effective antagonist to IL-6 signaling known as tocilizumab, is a monoclonal antibody. It is vital to diagnose initially and treat it accordingly in an affected patient [40].

## Therapeutic approaches for combatting cytokine surge

The treatment strategy must restrict the proceeding inflammatory cytokine production and renew the homeostasis in the host’s body. The potential treatment is based on anti-inflammatory drugs that can control inflammation. Treatment differs according to the infection encountered by the individual [80]. The potential treatment used for cytokine storm in COVID-19 is as follows:

### Etoposide

Etoposide is an inhibitor of topoisomerase II. In a mouse model of HLH, its curative strategy was demonstrated to entail substantial deletion of stimulated T cells and effective repression of inflammatory cytokine production. There was no apparent anti-inflammatory impact observed on macrophages or dendritic cells, nor was there any depletion of quiescent naive or memory T cells. Similarly, in a study, the mice infected by SARS-CoV, monocyte-macrophage inflammation responses eventually led to fatal pneumonia, recommending the vital role of inhibiting such monocytes in labeling severe pneumonia which is related to SARS-CoV to understand the consequences or effects that can have an impact on the patient due to the introduction of the drug [81, 82].

### Intravenous immunoglobulin (IVIG)

A therapeutic construction of highly refined, polyclonal IgG antibodies from the plasma of multiple donors. It is a treatment for people with weakened immune systems which can be given intravenously. Presumably, the effectiveness of IVIG is neutralizing phagocytes (neutrophils, monocytes, and macrophages) via FcγRIIIb and FcγRIIa, as well as the neutralizing impact that inhibits viruses from entering host cells, it is hypothesized that IVIG may reduce the inflammatory environment and control lung complications, although additional research is required to determine the detailed process of IVIG effectiveness in individuals suffering from COVID-19. Following the combination of prednisolone and intravenous immunoglobulin (IVIG), Th1 cytokines (IFN-γ, IL-2) and viral loads were recovered. According to reports with prior positive experiences from SARS patients, it was advised to use a high dosage of IVIG in patients with critical COVID-19 in the beginning stages of the illness [83, 84].

### Colchicine

Colchicine’s anti-inflammatory effects are varied. It inhibits neutrophil activities, including adhesion, mobility, and degranulation of lysosomes, as well as neutrophil chemotaxis. Colchicine inhibits neutrophil movement and cross-talk with endothelial cells and regulates the generation of pro-inflammatory cytokines such as IL-1, IL-6, and tumor necrosis factor (TNF)-α by reducing the levels of molecules that bind on neutrophil membranes [85]. Depending on its capability to inhibit the production of IL-1, colchicine is used in treatment. This has properties like anti-inflammation, which may be advantageous in alleviating the cytokine storm through the way it influences NLRP3 inflammasome; records show that colchicine can non-selectively take part in the inhibition of inflammation of NLRP3 by restricting the stimulation of the purinoceptor 7 (P2X7) receptor or the interaction between NLRP3 protein and ASC. Another benefit of using colchicine in treatment is the inhibition of IL-1β by disruption of the inflammasome complex, IL-6, and IL-18 stimulation. The major role of colchicine is to slow down the function performed by neutrophils [86].

### Tocilizumab

It has been noted from preceding records that IL-6 plays a vital part in immunogenic response. The accumulation of serum IL-6 increases more than normal level when an infection occurs. IL-6 is a specific arbitrator of inflammation and cytokine storm induced virally in COVID-19 patients. It is a humanized monoclonal antibody with an anti-IL-6 receptor and an aggressive inhibitor of the IL-6 receptors, inhibiting transduction pathways of IL-6. With the introduction of tocilizumab, elevated temperatures in all patients were solved on the initial day of administration. Though the molecular mechanism of the antibody is still indecisive, it has been disclosed that this drug helps improve critical COVID-19 patients. In many localities, this drug’s effectiveness is being evaluated [87]. A study reported that 21 individuals with severe cases of COVID-19 were selected from two institutions in China and given Tocilizumab treatment in lieu of standard medical treatment for COVID-19, which includes antiviral medication, oxygen therapy, and symptom relief. The subgroup treated with Tocilizumab demonstrated substantial reductions in clinical signs, including pulmonary function, fever, and oxygen therapy necessity. Additionally, CT scan outcomes demonstrated a reduction in lung opacities in 19 patients, and 19 out of 21 patients were released on average 13.5 days following therapy, whereas the remaining two patients stayed in steady condition; furthermore, no severe side effects related to tocilizumab were reported in the present research [88].

### Corticosteroid

One of the anti-inflammatory medications most frequently used to treat inflammatory conditions. It is majorly allocated to severe COVID-19-affected individuals. They exhibit the consequence of immunoregulation by hindering the articulation of various pro-inflammatory cytokines. Glucocorticoids minimize T cells and macrophages’ amplification, stimulation, and viability [89]. Further research indicates that the initial administration of steroids during infection with SARS-CoV is correlated with an elevated plasma viral burden and a delay in viral clearance [90]. Perhaps these drugs inhibit the systemic immune reaction correlated with ARDS. Nevertheless, existing empirical evidence indicates that morbidity and subsequent infection rates are rising in such patients receiving corticosteroid therapy [91]. WHO’s existing interim advice on the therapeutic treatment of severe acute respiratory infection triggered by SARS-CoV-2 strongly forbids the administration of corticosteroids unless otherwise suggested [92].

### Chloroquine/hydroxychloroquine

Chloroquine (CQ) was used in the treatment of malarial infection. Hydroxychloroquine (HCQ) is a less harmful metabolite of chloroquine. They possess properties like anti-inflammation that could overpower CS. Both have the potential to restrict the lysosomal activity and the function they carry out. When taken as a whole, it causes a reduction in the production of many pro-inflammatory cytokines, including IL-1, IL-6, and IFN, which is a mediating factor of the cytokine storm. Acknowledging that the two CQ and HCQ can exhibit a broad scale of action against COVID-19 by employing direct intervention with cytokine storm [93] is essential. In *vitro* investigations show that chloroquine and hydroxychloroquine suppress SARS-CoV-2 despite the latter being considerably more efficacious [27]. The suggested mechanism of action is that the drug inhibits the integration of host cells with the virus by elevating the endosomal pH.

Additionally, it inhibits the glycosylation of ACE2 receptors, preventing the viruses from getting into the intended cells [93, 94]. It has shown promising results in reducing SARS-CoV-2-induced pneumonia with advancements in pulmonary imaging observations and a shortened duration of the illness. However, based on the findings above, no strong confirmation is available to assume that CQ and HCQ possibly be productive and safe in treating patients with COVID-19 [95].

Despite the fact that the immunological and pathological understanding of COVID-CS has contributed essential knowledge for the advancement of diagnostic as well as therapeutic approaches, comprehensive directions still need to be provided. Developing scoring systems such as the MS score, H Score, the Penn grading scale, HLH-2004, and the Common Terminology Criteria for Adverse Events could be advantageous for predicting COVID-CS or its associated consequences. The study suggested the diagnostic criteria for COVID-CS. These criteria consist of three groups: (1) alanine aminotransferase > 60 IU/L, aspartate aminotransferase > 87 IU/L, D-dimer > 4930 ng/mL, lactate dehydrogenase > 416 U/L, troponin I > 1.09 ng/mL; (2) albumin < 2.87 mg/mL, lymphocytes < 10.2%, neutrophil absolute count > 11.4 × 103/mL; and (3) anion gap < 6.8 mmol/L, chloride > 106 mmol/L, potassium > 4.9 mmol/L, and blood urea nitrogen: creatinine ratio > 29. Furthermore, ferritin > 250 ng/mL and C-reactive protein (CRP) > 4.6 mg/dL are added to confirm active systemic inflammation [96]. In another study, the authors suggested that diagnostic parameters such as peripheral blood oxygen saturation in relation to the volume of inspired oxygen (SpO_2_/FiO_2_), ferritin, CRP, cytokines, and neutrophils ratio could indicate a high diagnostic ability for COVID-CS [97]. Similarly, another study suggested that all critically ill COVID-19 individuals should undergo an initial evaluation for hyperinflammation, implementing diagnostic tests and the H Score to detect COVID-CS [98]. Regardless of the need for additional evaluation, these parameters serve as valuable recommendations for facilitating the development of officially recognized specifications for COVID-CS.

## Blocking of signaling pathways

### TNFα blockade

TNF-α blockade is the potential strategy to reduce excessive cytokine release in COVID-19 individuals. Well-studied blocking agents of TNF-α are TNFR that are soluble and monoclonal neutralizing antibodies (mNAbs) as they prevent the communication between TNF-α and their receptors. Infliximab is a medically accepted blocker of TNF-α [99]. Adalimumab is an antibody that targets the blocking of TNF-α. The signaling pathway triggered by the binding of TNF-α with receptors includes NF-κB, which helps in the path. Sodium salicylate and aspirin inhibit NF-kB activation by hindering Ikappa B kinase [80].

### Blocking of IL-6 signaling

IL-6 signaling is known as the persuader of COVID-CS. IL-6 inhibitors, such as siltuximab, and IL-6R inhibitors, such as tocilizumab, are available pharmaceutical agents that specifically target the signaling pathway of IL-6. Reported trials revealed positive results of some IL-6 or IL-6R antagonists for treating cytokine storms in patients. Tocilizumab is a monoclonal anti-IL‐6R antibody. It attaches to both soluble and membrane‐bound IL‐6R to suppress IL-6-facilitated signaling. Studies on biomarkers are essential to understanding the risk and therapeutic effect for future use [100].

### Blockade of IL-1β

The clinical part of IL-1β signaling in CS is well known. Drugs that target the signaling of IL-1β, including IL-1β antagonist (canakinumab) and IL-1 receptor antagonist (anakinra), might be advantageous in treating COVID-19 CS. A Study in Italy suggested that introducing 300 mg canakinumab in affected patients rapidly decreased inflammation and enhanced oxygenation in COVID-19 patients [101].

### Blockade of IL-2

IL-2 is a known cytokine signaling molecule. It is fundamental for the immune system’s activation. IL-2 signaling is carried out through its receptor, a complex of hetero protein whose gamma chain is collectively used by IL-4 and IL-7. Therapeutic antibodies daclizumab and basiliximab are blocking antibodies that inhibit the IL-2 pathway and bring about partial immunosuppression [102, 103].

### Blockade of IL-7

IL-7 is a hematopoietic cytokine produced by stromal cells originating in the bone marrow. It produces lymphocytes through its receptor by promoting differentiation. IL-7 levels are escalated in affected patients. The M25 antibody is beneficial in nullifying the biological activity of IL-7 and its administration [104].

### Blockade of IL-10

A distinctive characteristic of the COVID-19 cytokine storm is the increased levels of IL-10 in critical patients. A possible target for decreasing COVID-19 mortality is IL-10 [26]. Using an antibody that blocks IL-10 to restrict the effect of immune activation in the beginning stage of COVID-19 might be beneficial to test. The scheduled time for suppressing the activity of IL-10 in critically ill patients may be important [105].

### Blockade of IL-12

IL-12 greatly aids Th1 and Th17 cell development. It also initiates NK cells. Inhibitors of IL-12 used in clinical traits are risankizumab, tildrakizumab (targeting IL-23p19), and ustekinumab (targeting IL-12/IL-23p40), mostly for chronic inflammation and autoimmune diseases. The reliability of case reports related to the inhibition of IL-12 is relatively weak, which yields the importance of randomized, controlled, prospective clinical trials [106].

### Blockade of INF-ϒ

**Fig. 3:**
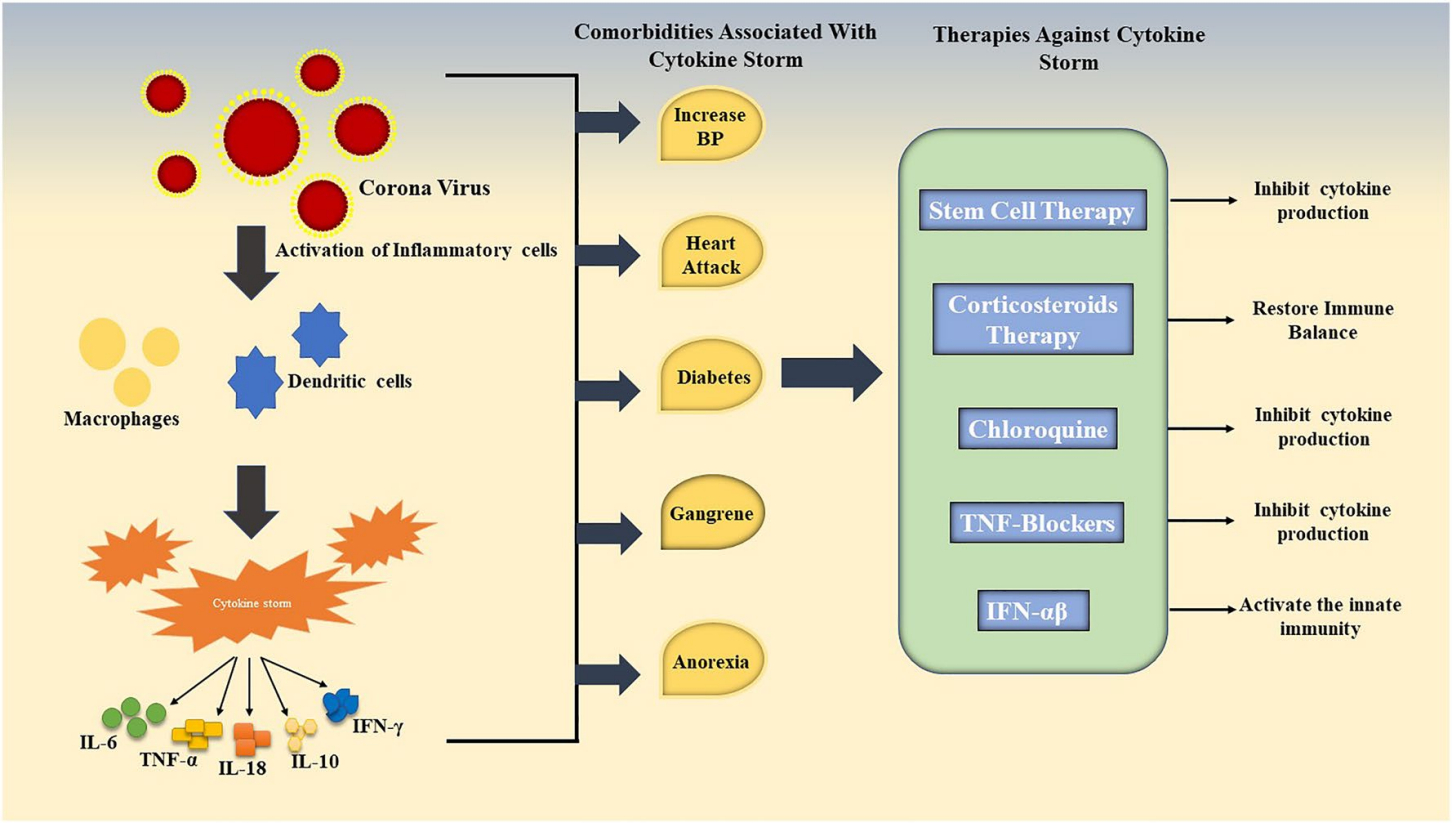
Picture representing the comorbidities associated with the CS and various potential therapeutic approaches against COVID-19 infection

INF-γ is an immune interferon produced mainly by T cells and NK cells when reacted with antigens. Emapalumab, an antibody that targets IFN-γ, is being authorized to be introduced in treating HLH, a condition with increased serum levels of IFN-γ_._ A clinical trial performed in Italy was enrolled to inspect the potency of emapalumab and anakinra to reduce increased inflammation and alleviate respiratory conditions in administered patients [95]. Figure 3 summarizes the comorbidities associated with CS and potential therapeutic approaches against COVID-19 infection.

## Potential for re-infection with COVID-19 and its variants

Currently, cases of re-infection with COVID-19 variants are on the rise. Some altered versions, like Alpha and Delta, can escape the immune system and spread more quickly. However, a version of Omicron that is less violent and whose re-infection rate is unknown has been found. All over the world, new versions of COVID-19 are causing a new wave of deaths and several cases of re-infection. Even though the number of re-infections has been significantly understated globally, viral genome sequencing is needed to prove re-infection. After all, the SARS-CoV-2 strains are different in primary and secondary infection. The high number of alterations in the spike glycoprotein (S-protein) of the Omicron version may help the virus avoid stopping antibodies and other immune reactions like the T-cell response. Initial analysis showed that the version has more than 30 alterations in the part of the virus that codes for the spike protein that lets the virus into human cells. Studies have also demonstrated that the Omicron variant was about 30 times more likely to get re-infected than the Alpha version and 10 times more likely than the Delta form [107].

## Global health governance strategies in managing COVID-19 and its Variants

When a person with serious COVID-19 disease recovers from the virus, it depends on how severe and to what extent the virus attacks different cell types and organs during the acute phase of infection. Post-COVID phase is often used to describe some people’s long-term effects after being infected with COVID-19. Some signs only last for a short period, while others are found to reoccur. In reaction to the pandemic, the new coronavirus forces the global healthcare government to make new decisions about policy. In this situation, some hospitals have set up “post-COVID day hospitals.” Most of the time, geriatricians are in charge of these settings because they have the knowledge and skills to deal with people with multiple health problems. Even though it is known that the Zika virus, Ebola, and HIV/AIDS sentinel outbreaks showed that the world’s “therapeutic geographies” were strongly shaped by racial histories, colonial legacies, and post-colonial geopolitics. COVID-19 was mainly managed with the help of biological solutions and new developments in data science, technology for finding contacts, and artificial intelligence (AI). Research and Development (R&D) is speeding up the production of pharmaceuticals so that in future, there will be better universal healthcare systems linked to social safety. The government has taken many steps to help the economy rebound. The pandemic has shown and sped up how the government changes its policies and thinks about them. It has also brought to light the adoption of many new ideas, which has led to varying degrees of changes in law enforcement, health care, and education. Governments need to take a regularizing approach when choosing and controlling long-lasting data technologies to avoid long-term side effects that are surprising and could be awful. In managing the post-COVID era, strategies like early public participation, dynamic consent, and improving digital literacy could help co-create visible, trustworthy, and real anti-epidemic technologies with mechanisms for transparency and accountability [108].

## Strategies for post-COVID management

Since well-designed randomized controlled trials are lacking for post-viral infections, it is challenging to develop an evidence-based approach for its therapy. Post-viral tiredness that lasts longer than six months may qualify as chronic fatigue syndrome or myalgic encephalomyelitis (ME) [109]**.** Since post-viral infection is a milder version of chronic fatigue syndrome (ME/CFS), it stands to reason that the same approaches used to treat ME/CFS might also be effective in treating post-viral infections. An estimated eighty percent of those who recovered from COVID-19 with just minor symptoms will show no residual effects and will fully recover. Patients who initially seemed to have acute symptoms and required hospitalization but ultimately did not require mechanical breathing did not have difficulties in the long run [110]. Patients with severe symptomatology who need breathing assistance have a higher risk of complications and an extended course to recovery [111]. Alterations in the pathophysiology of SARS-CoV-2, damages caused by inflammation, and immunological anomalies in COVID-19 are all possible paths that could result in post-COVID-19 problems. In severe COVID-19 survivors, the numerous multiorgan systems have the potential to be damaged [112]. COVID-19 survivors have reported a wide range of lung problems, from shortness of breath to a complex lung injury and ventilator support [112]. Post-COVID-19, corticosteroid medication may be beneficial for certain patients, as shown by a preliminary discovery of a significant radiological and symptomatic change in a sample of recovered patients [113]. Previous lung transplantation was performed for fibroproliferative lung disease, which is similar to ARDS induced by severe COVID-19 and influenza A (H1N1) [112]. Clinical investigations aimed at suppressing lung fibrosis after COVID-19 include the use of antifibrotic medications [114]. Venous thromboembolism (VTE) was found in 5% of the COVID-19 patients who got better. Even though there isn’t enough proof to say for sure, people with SARS-CoV-2 infections who get primary thromboprophylaxis for up to 45 days, stay in the hospital longer (up to 6 weeks), and are treated as outpatients may have a better risk-to-benefit ratio [112]. Infections after COVID-19 are best treated with anticoagulants like straight oral anticoagulants and low-molecular-weight heparin [115]. Anticoagulation drugs are given to people with venous thromboembolism that has been proven by imaging (up to 3 months) [116, 117].

Individuals who do not have a pre-existing diagnosis of diabetes mellitus have seen the onset of diabetic ketoacidosis many weeks to months following the resolution of COVID-19 symptoms [118]. The COVID-19 pandemic has the potential to worsen autoimmune thyroid disorders, including Hashimoto’s thyroiditis and Graves’ disease [119, 120]. For individuals who have recently been diagnosed with diabetes mellitus and lack the common risk factors associated with type 2 diabetes, it is recommended to conduct serologic tests to detect type 1 diabetes-related autoantibodies and repeat post-prandial C-peptide analyses during follow-up. Conversely, for patients who possess these risk factors, it is advisable to manage their condition as if they were experiencing ketosis. Individuals have an increased susceptibility to developing type 2 diabetes. [121]. Corticosteroids have been shown to be effective in managing hyperthyroidism that arises from disruptive thyroiditis associated with SARS-CoV-2. [122].Corticosteroids have been shown to be effective in managing hyperthyroidism that arises from disruptive thyroiditis associated with SARS-CoV-2 [123, 124]. The ethmoid sinuses have the highest frequency of affliction, with the maxillary sinus following suit [125]. The research reveals that a notable proportion of secondary bacterial or fungal infections, namely 8%, emerged among individuals who were either COVID-19 patients or had recovered from COVID-19 during their hospitalization. This occurrence persisted despite the administration of steroids and antibiotics in a comprehensive manner [126]. Once the diagnosis is made, the fungus-infected area should be cleaned up by surgery as soon as possible. To start, amphotericin-B deoxycholate is the best antifungal treatment, and liposomal forms are better because they are less harmful to the kidneys. Posaconazole is a good option for amphotericin when it doesn’t work or doesn’t work well enough. As a result, managing the aftermath of COVID necessitates taking a comprehensive strategy that considers not only the body but also the mind and society. People and communities may go on the road to recovery and resiliency if they combine several types of help, such as medical treatment, psychological counseling, and social adjustments. It is essential to place a high priority on research, education, and cooperation in order to guarantee that the lessons learned from the pandemic will contribute to the construction of a future that is both healthier and more equipped.

## Innovative approaches for post-COVID management

The persistent struggle against the COVID-19 pandemic necessitates a constant need for adjustment and ingenuity in implementing management tactics aimed at mitigating the spread of the virus, lessening the severity of its impact on individuals, and finally gaining control over the outbreak. The ongoing presence of COVID-19 highlights the need for novel approaches that surpass conventional preventative efforts. The area of science has been enriched by several advancements, such as the identification of novel pharmaceuticals and vaccines, the repurposing of existing medications, the utilization of in vitro drug screening methods, the implementation of in silico screening platforms, the creation of innovative technologies, and the introduction of fresh diagnostic procedures. AI is a rapidly growing field of study that offers novel avenues for research and development, particularly in the realm of medication discovery and development [127]. The process encompasses the timely identification and assessment of the infection by the use of CT scanning, X-ray imaging, laboratory testing, and genome sequencing. The aforementioned research demonstrates a significant potential in the field of drug discovery, namely in the development of new medications and the repurposing of existing ones, with the aim of effectively treating COVID-19. The study employs many datasets to find putative actions that suppress human coronaviruses. In recent times, there has been an observed decrease in the quantity of newly authorized pharmaceuticals by the Food and Drug Administration (FDA) owing to the presence of adverse effects and diminished efficacy associated with several medications [128, 129].

Repositioning of the drug is another name for drug recycling. It is a process that tries to find new uses and applications for current drugs. It is thought to be a cost-effective method. [130, 131]. The emergence of the COVID-19 pandemic has presented significant challenges to healthcare professionals, researchers, and chemists in identifying an effective pharmaceutical intervention. However, developing novel vaccines and drugs has proven to be a resource-intensive and time-consuming process, with a success rate of merely 2.01%. Consequently, in light of the associated costs and time constraints, the concept of repurposing existing drugs for the treatment of COVID-19 has gained tract [130]. Drug repurposing is an organized process that can be broken down into theoretical and experimental methods [132]. Lopinavir, hydroxychloroquine, atazanavir, nintedanib, tocilizumab, and remdesivir are some of the drugs used to treat COVID-19 at different levels [133, 134]. Recent clinical studies have shown that the administration of nintedanib in patients with COVID-19 results in a reduction in the production of IL-1 and IL-6. These cytokines are known to play a significant part in the development of the COVID-19 cytokine storm, which subsequently leads to the formation of fibrous tissue in the lungs [135]. COVID-19 patients with IPF are prescribed it because it represents a potentially effective new method of treating COVID-19 [136]. The 80:20 blend of the equipotent homologous 22,23-dihydro ivermectin is what makes ivermectin the FDA-approved anti-parasitic medicine that has been demonstrated to suppress SARS-CoV-2 in vitro with an unknown mechanism of action [137]. Researchers conducted comprehensive computer analyses to determine which ivermectins docked most well with viral proteins and, subsequently, to examine possible structural changes using molecular dynamics. Ivermectins may bind to the HR2 domain and the surface and internal pockets of the 3C-like protease (3CL), changing its natural conformation [138]. Chloroquine (CQ), an aminoquinoline, has been conventionally used for the treatment of malaria using chloroquine phosphate over an extended period of time. The compound in question is a member of the quinolone class of drugs, exhibiting anti-inflammatory and amoebic effects. The success of chloroquine in treating individuals with COVID-19 may be attributed to its antiviral and anti-inflammatory properties [139].

In the context of a growing pharmaceutical industry, it is essential to emphasize the significance of cooperation between pharmaceutical companies and regulatory bodies. The regulatory agency is responsible for ensuring the safety, effectiveness, quality, and performance of medical items. The primary responsibility of national regulatory authorities is to ensure that medicines and biological products adhere to established standards of quality and safety. There is also a worldwide training network in place to address gaps in vaccination quality and provide assistance in developing programs. In the COVID-19 age, one of the new fields is nanotechnology. It is used to find new drugs and opens up new possibilities for cheap ways to find COVID-19 and make medicines against it. Several nanomaterials can be used to stop COVID-19, like putting nanomaterials into kits of personal safety equipment for health workers to keep them safe. Nanoparticles that target ACE receptors can stop virus particles from attaching to cells. Nanoparticle-based medicines and drug delivery methods can also be used to control SARS-CoV-2 [140]. The use of nanosensors and nano-filter face masks has been identified as a potential strategy for mitigating the transmission of infectious illnesses [141]. In recent years, there have been advancements in the area of biosensing using field effect transistors (FETs) for the purpose of infection detection. Various nanomaterials, such as carbon black, graphene, gold, and silver particles, are used in biosensors to facilitate the detection of SARS-CoV-2 infections [142]. Further investigation is necessary to ascertain the potential utilization of nanotechnology during the current pandemic. It is essential to promote safety protocols while simultaneously expediting the use of nanoparticles for the treatment of COVID-19. Viruses infiltrate the human body via the nasal passage upon inhalation. Consequently, scientists worldwide are directing their efforts toward the development of vaccinations that may effectively counteract the aerosols responsible for nasal invasion. The intranasal vaccination has the ability to intercept the virus and stimulate the immune response to effectively battle viral infections at the point of viral entrance. This mechanism of action effectively prevents the proliferation of viruses inside the body [143]. Nasal vaccines are characterized by their ease of administration and ability to induce the production of secretory IgA, hence facilitating an immune response at the specific site of viral entry [144, 145]. A recent study conducted in India examined the efficacy of a nasal vaccine compared to mRNA and traditional vaccines. The findings revealed that the nasal vaccine exhibited a significant reduction in viral load replication, effectively preventing inflammation and pneumonia when compared to other vaccine types. Additionally, the nasal vaccine demonstrated superior neutralization capacities. The latest clinical trial findings indicate that a nasal vaccination containing nitric oxide is safe and efficacious in suppressing SARS-CoV-2 infection. In their investigations on murine subjects, King et al. discovered that the administration of a solitary intranasal vaccination dosage may induce immune responses in both the mucosal and systemic compartments. Several nasal vaccines undergoing phase I and phase II studies include ChAdOx1 nCoV-19, Ad5-nCoV vaccination, NasoVAX, DelNS1-nCoV-RBD LAIV, and Mv-014–212 [143]. The dynamic nature of the COVID-19 epidemic necessitates the adoption of a proactive and inventive management strategy. Through the integration of breakthroughs in vaccines, antiviral medicines, data-driven interventions, personalized testing, and effective communication, society may effectively address the issues presented by the virus. The pursuit of ongoing research, adaptability in the execution of strategies, and international cooperation are crucial in attaining enduring management of the pandemic and fostering a more resilient global society [127].

## Alternative therapeutic strategies for post-COVID management

Prevention stands as a vital step in the battle against the COVID-19 pandemic. The use of pre-exposure preventative measures might be regarded as a primary approach in combating this illness. The prevention of viral entrance and replication is of utmost importance [146]. The use of naturally occurring chemicals can serve as an alternative method of prophylaxis by enhancing the immune response during the pre-exposure phase. Plant-based diets have been shown to have a positive impact on the composition and function of beneficial bacteria in the intestines, hence promoting the general health of the gut microbiome. It is worth noting that the gut microbiome constitutes around 85% of the body’s immune system [147]. Curcumin, the primary phytochemical derived from *Curcuma longa* L. exhibits antiviral properties against SARS-CoV-2, antioxidative capabilities, and possible immune-enhancing characteristics. It has strong antioxidant properties and can induce the synthesis of interferons, triggering the activation of the innate immune response in the host organism [148]. Piperine, an alkaloid compound derived from *Piper nigrum* L., has been found to enhance innate immunity by promoting the phosphorylation of interferon regulatory factor 3 (IRF-3) and IFN-1 mRNA. Additionally, piperine has been shown to inhibit the lipopolysaccharide (LPS)-induced expression of IRF-1 and IRF-7 mRNA, as well as reduce STAT-1 activity and the phosphorylation of IRF-3 and type 1 IFN mRNA [146, 149]. Garlic, which has also been proposed as a preventive measure, has the potential to alleviate symptoms in those who have been infected. The control of immunoglobulin production, phagocytosis, macrophage activation, and cytokine release leads to the stimulation of NK cells, lymphocytes, eosinophils, and macrophages [150]. Furthermore, garlic has been shown to possess the capacity to enhance the immune system by substantially augmenting the population of CD4^+^ T cells and the overall count of white blood cells [148, 151]. Aloe vera, scientifically known as *Aloe barbadensis miller*, is a perennial succulent plant belonging to the family Asphodelaceae*. Aloe vera* (L.) Burm. f. gel and its primary components, aloin, and aloe-emodin, have been identified as promising antiviral drugs. These chemicals have antiviral activity against enveloped viruses, including SARS-CoV-1, HIV, and influenza viruses, via mechanisms that include inhibition of viral replication or disruption of the viral lipid envelope [152, 153]. The aforementioned characteristics provide aloe a compelling selection as a primary botanical component in a non-alcoholic hand sanitizing formulation [154]. An in silico investigation was carried out on the essential oil derived from *Ammoides verticillata* (Desf.) Briq. which notably defined that the primary constituent of this essential oil, isothymol, exhibited promising outcomes when evaluated in the context of the isothymol-ACE2 docked complex [155]. The aforementioned component is also present in ajowan essential oil, which is derived from the aerial portions of the plant. This essential oil has been shown to possess antiviral and antibacterial effects [49]. Comparably, the utilization of various methodologies such as docking, absorption, distribution, metabolism, and excretion (ADMET) properties calculation, molecular dynamics, and molecular mechanics/Poisson–Boltzmann or generalized born and surface area (MM-PBSA) approaches have provided insights into the strong binding affinity of dithymoquinone (DTQ), an active compound found in *Nigella sativa* L. to ACE2 [156, 157]. The inhibitory effects on ACE2 have also been seen in the components of propolis, which is a resinous material synthesized by bees [157]. Quercetin, a flavonol compound belonging to the polyphenolic flavonoid group, may be found in a diverse range of dietary items such as grapefruit, onions, apples, and black tea. Additionally, it is also present in some plants, including *Hypericum perforatum* L*.* and *Sambucus nigra* L. [147, 158]. The suggested intervention exhibits potential efficacy in disrupting the binding of viruses. Nevertheless, it is important to acknowledge that the efficacy of this drug, when administered orally, is improbable owing to biotransformation. Consequently, it is recommended to be used as a nose or throat spray [159]. *Cathepsin* L. (CatL), an enzyme located in the lysosomes of endosomes, plays a significant role in several physiological and pathological processes. These processes include extracellular matrix remodeling, antigen processing, apoptosis, invasion, inflammatory state, and viral infection. CatL plays a significant role in the degradation of the extracellular matrix, which is a crucial mechanism for binding the SARS-CoV-2 spike protein and subsequent entry into host cells [160]. Hence, the identification of plants and phytochemicals that possess the ability to inhibit CatL might be regarded as a significant therapeutic objective for the management of individuals afflicted with COVID-19. In a study, screening was performed to identify potential inhibitors of CatL. The reproduction process of some viruses, such as SARS-CoV, requires the presence of cyclophilin [161, 162]. There exists empirical data supporting the capacity of Cyclosporin A, a pharmacological agent that inhibits cyclophilins, to effectively impede the reproduction process of coronaviruses [163]. As far as current literature is concerned, there is a lack of research pertaining to the suppression of cyclophilin by medicinal plants. Nevertheless, much research has been undertaken to examine the inhibition of coronavirus replication in plant organisms [164, 165]. Glycyrrhizin, the primary bioactive component found in liquorice root, is a molecule of significant importance due to its diverse antiviral properties, notably its potent ability to inhibit the reproduction of SARS-CoV [162–164]. Moreover, it has been shown that *Nigella Sativa* L, *Anthemis hyalina*, and citrus sinensis have noteworthy impacts on the expression of transient receptor potential (TRP) genes and the viral load. Among these, *A. hyalina DC* has been found to possess the highest potency in this context [162]. The use of natural resources and medicinal plants has significant potential in the realm of medication development for the effective management of COVID-19. In line with the methodologies used in the development and manufacturing of semisynthetic and synthetic drugs, it is essential to adopt a systematic and logical approach. Natural products have the potential to provide both preventative and therapeutic assistance in mitigating the effects of the pandemic. These tactics may be used by researchers in the challenging circumstances of medication development for combating COVID-19. This opportunity can potentially contribute to the prevention and management of the COVID-19 pandemic. Indeed, a considerable number of investigations have been carried out throughout the pandemic, focusing on natural products. However, it is worth noting that a significant portion of this research has been completed using in silico methods. It is advisable to conduct future research works in order to validate the efficacy of these medicinal plants, as well as to investigate novel botanical remedies.

## Discussion

The CS during infection can lead to complications in our body due to the overexpression of the cytokines, and inflammatory cytokines can even act as a therapeutic target in future [19]. The accumulating cytokines form a storm, which aids in the spread of SARS-CoV-2. As a result, cytokines are released more often, triggering a hostile inflammatory response. Even ARDS has been linked to and induced by high levels of cytokines such as IL-8, IL-6, and IL-1. As a result, the generated cytokine storm triggers an inflammatory response that is said to be a contributing cause of COVID-19 mortality. As a result, focusing on the increased cytokines and other pathways that enhance the production of the cytokines persistently is an appealing strategy for eradicating one of COVID-19’s trademarks and, ideally, lowering COVID-19 mortality rates [26].

Furthermore, immunological memory provides the basis for durable immune defense in the wake of diseases or immunizations. How long the immune response to SARS-CoV-2 or COVID-19 will last is unknown. Memory B cells, antibodies, CD4^+^ T cells, and CD8^+^ T cells are all possible components of immunological memory. Understanding the protective immune response against COVID-19 and the likely course of the COVID-19 pandemic requires an appreciation of the immunological memory of SARS-CoV-2. Significant immunological memory, including the four primary forms of immune memory, is produced after a COVID-19 infection. According to research, over 95% of individuals kept their immunological memory for at least 6 months after infection. The levels of antibodies in the blood were shown to be unreliable indicators of T-cell memory. Therefore, serological tests for SARS-CoV-2 antibodies are inadequate in measuring the breadth and durability of SARS-specific immunological memory. Thus, the lack of a discernible half-life at 5 to 8 months post-infection, memory B lymphocytes specific for the spike protein or receptor-binding domain (RBD) have been detected in virtually all instances of COVID-19. Similar findings have been drawn from other receptor-binding domain memory B-cell studies. Different research describes the SARS-CoV-2 T-cell memory after 6 months. Researchers have estimated that smallpox-resistant memory CD4^+^ T cells have a lifespan of 10 years, consistent with the most recent observation of SARS-CoV-specific T cells about 17 years after the initial encounter with the virus [166]. These results suggest that T-cell memory may achieve a better equilibrium or a delayed breakdown phase during the first eight months after infection.

Evidence has shown the presence of significantly elevated levels of IL-6 in individuals diagnosed with COVID-19 [95]. Few drugs like tocilizumab, sarilumab, and siltuximab have therapeutic potential during this complication in patient physicians [167]. The drug chloroquine/hydroxychloroquine has shown significant anti-inflammatory properties that reduce the effect of interleukins. Hence, it was widely used as a treatment for COVID-19 [95]. Potential therapeutics like corticosteroids and using drugs obtained from the combination of antiviral agents or immunosuppressant’s are seen to bring immense relief to patients and physicians in future [168]. The post-COVID management strategies encompass a range of approaches aimed at supporting individuals recovering from the effects of the disease. The techniques encompass several aspects of care, such as physical and respiratory rehabilitation, psychological support, dietary advice, and continuous medical monitoring, to facilitate a holistic healing process. In the context of formulating novel methodologies for the management of COVID-19 infections, there is a growing interest in investigating alternative techniques such as monoclonal antibody treatments and immunomodulatory medicines. These approaches aim to specifically target certain facets of the illness, such as the cytokine storm. Furthermore, it is worth exploring alternate approaches to treat COVID-19 patients along with prescribed drugs, which helps the examination of non-pharmaceutical therapies, including the potential impact of plant-based diets in moderating the spread and severity of COVID-19 infection. These public health interventions, in conjunction with vaccination initiatives, have the potential to effectively manage the transmission of the virus and mitigate its consequences on communities. In general, the integration of post-COVID management tactics, inventive treatment approaches, and alternative preventative measures together enhance the comprehensiveness and efficacy of the response to the persistent issues presented by the COVID-19 pandemic.

## Conclusion and future perspectives

The series of events that take place to cause cytokine surge in COVID-19 patients are responsible for the inflammatory responses that can be life-threatening. The patients undergo complications like multiorgan failures, ARDS, and death. Immune dysfunction plays a vital role in the significant increase of cytokines. Mortality in patient is caused by an excessive immune response, which is facilitated by the production of several inflammatory cytokines (such as IL-12, IL-6, and IL-2) and chemokines (including CCL2, CCL3, CXCL10, and CCL4). Understanding the signaling pathways of the inflammatory cytokines and chemokines has provided insight into performing the blockade of the various signaling pathways. An increased amount of IL-6 has been noted in non-survivors. The specific treatment for the cytokine surge is still unknown, but the treatment provided is to reduce inflammation. Treatment using etoposide, colchicine, intravenous immunoglobin, corticosteroids, and chloroquine/hydroxychloroquine have reduced inflammation. Considering the risk of re-infection to understand the duration of protection and risk of re-infection and recurrence after natural infection is decisive for scheduling COVID-19 vaccination for individuals at risk, particularly with the emergence of more transmissible variants. The potential treatments and further understanding of the cytokine surge in future by researchers and physicians can provide advanced and specific treatment for patients. In the management of the post-COVID era, strategies such as early public participation, dynamic consent, digital literacy improvements, and the appointment of third-party judicial could be considered to facilitate the co-creation of noticeable, trustworthy, and genuine anti-epidemic technologies with mechanisms for transparency and accountability. Thus, it is essential to be well informed on the most recent updates on COVID-like illnesses and diligently follow public health guidelines. This is crucial in safeguarding individual well-being as well as the overall health of the community. The research outcomes summarized by other researchers and clinicians in this review may help researchers stay updated on ongoing research on COVID and its clinical management strategies.

## Data Availability

Data sharing is not applicable to this article as no datasets were generated or analyzed during the current study.
